# Wartime toxicology: the spectre of chemical and radiological warfare in Ukraine

**DOI:** 10.1080/24734306.2022.2056374

**Published:** 2022-04-01

**Authors:** P. R. Chai, Y. Berlyand, E. Goralnick, C. E. Goldfine, M. J. VanRooyen, D. Hryhorczuk, T. B. Erickson

**Affiliations:** a Department of Emergency Medicine, Mass General Brigham, Division of Medical Toxicology, Brigham and Women’s Hospital, Boston, MA, USA; b Department of Psychosocial Oncology and Palliative Care, Dana Farber Cancer Institute, Boston, MA, USA; c The Koch Institute for Integrated Cancer Research, Massachusetts Institute of Technology, Cambridge, MA, USA; d The Fenway Institute, Boston, MA, USA; e Center for Global Health, University of Illinois College of Medicine, Chicago, IL, USA; f Harvard Humanitarian Institute, Boston, MA, USA

**Keywords:** Chemical weapons, CBRN, Ukraine, Russia, radiation, nerve agents

## Abstract

The unprovoked invasion of Ukraine by the Russian Federation has resulted in the largest humanitarian crisis in Europe since World War II. As fighting intensifies throughout Ukraine, there is an increasing concern that the Russian Federation may consider the direct use of chemical or radiological weapons against military personnel and civilians in Ukraine. Despite prohibition of chemical weapons from the Chemical Weapons Convention of 1997, recent evidence has demonstrated that state actors will continue to use these agents as weapons of war and terror, despite publicly denying their use. We review chemical weapons produced and used by the Russian Federation (or its allies) to identify plausible risks in the Russian war in Ukraine. We also provide rapid assessment and treatment guidelines to recognize and manage these acute exposures.

## Introduction

In the early hours of February 24, 2022, Russian forces invaded Ukraine sparking an ongoing conflict of devastating morbidity and mortality [1]. During the first weeks of fighting, nearly three million Ukrainian civilians have been displaced from their homes, and indiscriminate bombing has caused significant destruction of infrastructure in many cities in Ukraine [2]. Ukrainians have been forced find shelter or flee to western Ukraine and neighboring countries like Poland and Hungary [3]. The true toll of the invasion is likely much greater than what has been reported in the media. As the world watches a deadly invasion, several disturbing incidents like the capture of the Chernobyl power plant and dangerous firefight near Zaporizhzhia (the largest nuclear power plant in Europe) highlight the potential for a significant toxicological disaster amidst the war in Ukraine [4]. Additionally, given a history of confirmed and alleged chemical weapons development and attacks by the Russian government over the past decade, the spectre of unconventional chemical weapons attacks on opposing militaries, civilians, and governments, is concerning [5]. As toxicologists and emergency healthcare providers, we prioritize preparedness measures domestically, but the application of these concepts during war and conflicts internationally is equally important. In this commentary, we seek to describe potential classes of pharmaceuticals, chemical compounds, and radiological agents that may be used as weapons of war by the Russian Federation as evidenced by recent conflicts [6]. In addition to discussing the prior uses by the Russian Federation, we describe practical approaches to managing these toxicological emergencies in the context of war. We propose a rapid assessment, triage, and treatment guidelines to describe emergency management of potential toxicological exposure in wartime settings.

## Weaponized fentanyl analogs

In 2002, a group of Chechen terrorists attacked the Dubrovka theatre in Moscow, Russia, resulting in a hostage standoff [7]. In response, special forces under direction of President Putin instilled a chemical aerosol into the vents of the theater incapacitating those inside. Analysis of clothing from two victims and urine from another confirmed the presence of remifentanil, carfentanil and its metabolite norcarfentanil, suggesting a mixture of aerosolized opioids were used to incapacitate all persons inside the theatre [8]. Approximately 131 of 850 hostages died as a direct result of gas exposure [9]. While impractical on the battlefield, it is plausible that the Russian military could use similar compounds to cause significant casualties among opposing soldiers in closed spaces or civilians trapped in apartment blocks, hospitals, subways, or bomb shelter complexes. Based on previous operations by the Russian Federation, aerosolized opioids may be combined with inhalational anesthetics like halothane and pumped into enclosed locations [10].

**Figure 1.**
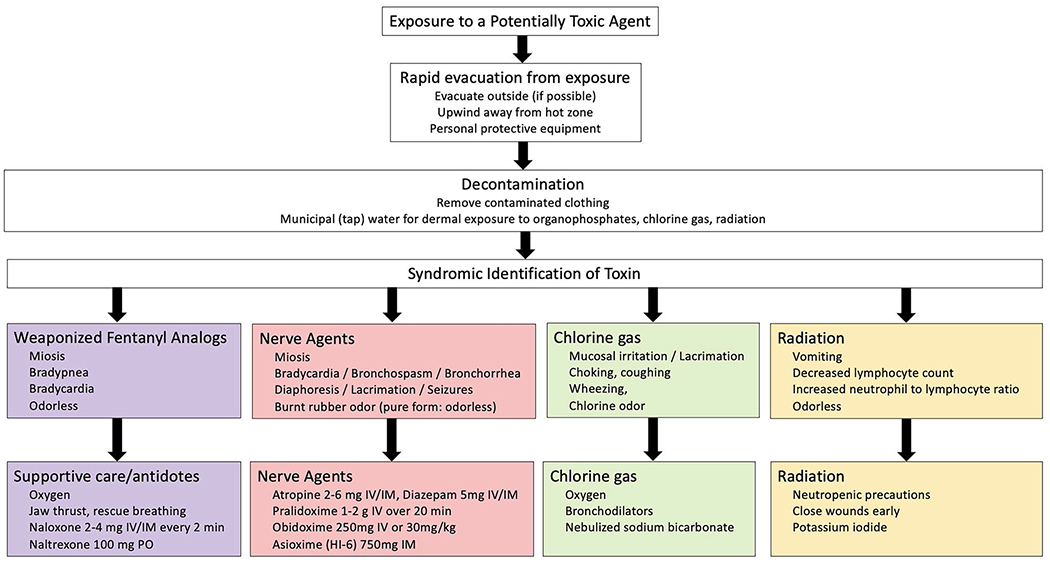
Rapid identification and treatment of potential chemical or nuclear weapons of war.

Opioid poisoning results in a classic opioid toxidrome of miosis, respiratory depression, and hypothermia. Death occurs from hypoxia and respiratory arrest. Management of opioid poisoning should focus on providing supportive airway management and rapid administration of opioid antagonists to reverse respiratory arrest (Figure 1) [11]. Naloxone, a muopioid receptor antagonist, is highly effective in reversing poisoning and can be administered intranasally, subcutaneously, intramuscularly or intravenously (IV) [11]. Treatment algorithms typically recommend administration of 0.04 mg naloxone IV in rapidly escalating doses until spontaneous respiration >12 breaths per minute is achieved. High potency fentanyl analogs may require larger or repeated doses of naloxone (2-4 mg through any available route). In individuals who may be potentially exposed to an aerosolized incapacitating opioid, clinicians may consider prophylaxis with oral naltrexone (100 mg up to 24 h prior to expected exposure) as an opioid block to prevent onset of respiratory depression [12–14]. This strategy has worked in opioid naive individuals in clinical trials but, to our knowledge, has not been attempted on the battlefield or in conflict settings. Clinicians and first responders should consider potential risk of persistence of this opioid block in the event of traumatic injuries which may need opioid analgesia. Additionally, this technique may also precipitate opioid withdrawal if inadvertently used in individuals who are opioid dependent.

## Chlorine gas

Since the battle of Ypres in World War I in 1915, chlorine gas has been used as a chemical incapacitating and asphyxiating agent [15,16]. Gaseous chlorine is a pulmonary irritant with a characteristically strong odor making it readily detected [17]. Its vapor density of 3.214 (air = 1) causes it to descend into underground spaces (e.g. metro stations, bomb shelters, etc.) to asphyxiate persons sheltering within them [18]. In 2018, the Assad regime in Syria used chlorine gas to devastating effect against civilians in Saraquib, a city south of Aleppo [19,20]. Given the simplicity of using chlorine gas and allegations that the Russian Federation supported the Assad regime in the assembly and deployment of these weapons, it is plausible that chlorine gas could be used in the ongoing war in Ukraine to incapacitate warfighters and cause morbidity among civilians. Potential delivery mechanisms include dropping canisters of chlorine gas from aircraft to cause incapacitation in anticipation of a ground attack, or combination of chlorine gas with an incendiary mortar to penetrate infrastructure. Chlorine gas toxicity results in pulmonary injury to the upper and lower airways. At concentrations around 1-3 ppm chlorine gas may cause ocular and oral mucosal irritation while a concentration of 15 ppm may result in pulmonary symptoms [21]. At high concentrations of approximately 430 ppm, death may occur within 30 min.

Exposed individuals will experience lacrimation, shortness of breath, bronchospasm, and wheezing (Figure 1). In enclosed spaces without ventilation, chlorine gas can persist and cause death from asphyxiation. Treatment of chlorine gas exposures focuses on evacuation from the environment. In exposed outdoor areas, dissipation of chlorine gas will result in resolution of symptoms. Individuals who are otherwise healthy without pulmonary disease will typically recover spontaneously over several hours. Victims with persistent wheezing or cough may be treated with supplemental oxygen, bronchodilators, or nebulized sodium bicarbonate [22]. Steroids have no clear role in treating lung injury from chlorine gas. When exposed to concentrations between 25-50 ppm, delayed pulmonary edema may occur within 2-4 h, and pulmonary mucosal sloughing may lead to secondary bacterial infections and fibrosis over several days [23].

## Nerve agents

Russian chemical weapon research has focused on the development of highly potent weaponized organophosphates, colloquially known as “nerve agents.” Despite a ban by the Organization for the Prohibition of Chemical Weapons (OPCW), sarin, VX, and *novichok* have seen use by Syria, North Korea, and Russia within the past decade. Kim Jong Nam rapidly died after exposure to VX orchestrated by North Korean intelligence agents [16]. Sergei and Yulia Skripal in 2018 and Alexei Navalny in 2020 are the best known victims of the Russian *novichok* agent [5]. Nerve agents generally are oily and persist on a variety of surfaces and materials. Skin absorption or inhalation will cause life-threatening symptoms [24]. Syrian government forces – supported by the Russians –repeatedly attacked civilians with sarin nerve gas and killed as many as 1,400 civilians in a single attack [20].

Nerve agents bind the acetylcholinesterase enzyme in the synaptic junction of central and peripheral neurons resulting in a characteristic cholinergic toxidrome: bronchorrhea, bradycardia, bronchospasm, miosis, diaphoresis, lacrimation, diarrhea and seizures (Figure 1). Death occurs acutely *via* seizures or asphyxiation from bronchorrhea and bronchospasm. Immediate treatment of nerve agent poisoning includes decontamination, removing victims from the exposure site, and early antidotal therapy [25]. The strong persistence and hydrophobic nature of nerve agents, removal of clothing from individuals and decontamination prior to entering a triage area can prevent inadvertent exposure of other patients and first responders to the agent. We recommend administration of diazepam in 5 mg increments to control seizures, and intravenous atropine 2-6 mg every 5-10 min until bradycardia and bronchospasm resolve [5]. Alternative sources of atropine may include veterinary hospitals or ophthalmic atropine that can be administered intramuscularly or intravenously. Intravenous or intramuscular pralidoxime 1-2 g (or other oximes like obidoxime or asoxime) given early can restore the acetylcholinesterase enzyme, but delays in oxime treatment reduce its effectiveness [26]. If atropine becomes scarce in a mass casualty event, other anticholinergic drugs including IV diphenhydramine, IV glycopyrrolate (glycopyrronium), or inhaled ipratropium will reduce bronchorrhea and bronchospasm [27,28].

## Radiation exposure

The 1986 reactor meltdown at Chernobyl highlights the risks to people and the environment in the event of a breach of the containment vessel. Since the explosion and reactor fire at Chernobyl, much of the surrounding environment has become a nuclear exclusion zone that will be uninhabited for centuries. The recent attack and fire at the Zaporizhzhia nuclear power plant and the loss of electrical power at Chernobyl highlight these risks [4]. Damage to a nuclear power plant due to intentional or unintentional strikes, or disruption of electricity or pumps to the water supply that is used to cool spent radioactive fuel rods could result in a catastrophic event similar to the Fukushima, Japan disaster [29]. In addition to acute radiation syndrome among those in the immediate vicinity, the release of radionuclides like radioiodine and radiocesium could cause widespread environmental contamination and increases in neoplastic diseases, especially thyroid cancer and leukemia.

Outside of exposures from fallout around nuclear power plants, pre-existing radioactive water storage facilities in East Ukraine used during the Cold War for the development of nuclear arms poses a unique source of chronic radiation exposure to the environment. Damage to these underground facilities may result in leakage of radioactive water into the water table, leading to contamination of agricultural fields and increases in background radiation exposure for inhabitants in these regions for decades [30,31]. Over two-thirds of mines in eastern Ukraine are at risk of compromise as a result of structural damage and natural flooding since 2014 during the war in Donbas, and the current Russian invasion [31]. Yuniy Komunar mine was the site of intense nuclear testing in 1979, and is still filled with radioactive water [30]. Oleksandr-Zakhid mine in Horlivka has been used since 1989 to store chlorobenzene and other carcinogenic toxins.

Acute exposure to high doses of ionizing radiation leads to the characteristic acute radiation syndrome (ARS). Acute exposures lead to the characteristic acute radiation syndrome (ARS). ARS is divided into three phases: prodromal phase (0-2 days after exposure), latent phase (2–20 days after exposure), and manifest phase ARS (21-60 days after exposure) [32]. The prodromal phase is characterized by nonspecific symptoms such as nausea, vomiting, diarrhea, fatigue, fevers, and headache. In acute, high-level exposures, death may occur through radiation poisoning of multiple cell lines manifest as bone marrow suppression, severe gastroenteritis, cardiac arrhythmias, and radiation induced neurotoxicity in the form of acute altered mental status and seizures. Radiation exposure to the skin can result in radiation burns presenting as erythema, inflammation, or desquamation. During the latent phase some patients experience an improvement in symptoms from the prodromal phase, although patients with severe exposure may progress directly to the manifest phase. During manifest ARS patients experience bone marrow suppression with resultant infections, anemia, and bleeding, GI manifestations with severe diarrhea and electrolyte abnormalities, cutaneous findings such as alopecia, desquamation of the skin, and finally cerebral edema and death. The release of radionuclides from a damaged reactor can travel far distances downwind and result in both internal radiation exposure from ingestion and inhalation of radionuclides and to external radiation exposure from the passing cloud. Based on the experience from the Chernobyl reactor accident, the greatest risk is from thyroid cancer caused by exposure to radioiodines. Depending on the proximity to population centers, this can result in exposure to millions of people, with children being at highest risk for development of thyroid cancer. Population exposure can be mitigated through sheltering, evacuation, and use of prophylactic potassium iodide, though these countermeasures are extremely difficult in wartime.

Radiation exposure requires rapid identification of the dose, rapid external decontamination, and triage (Figure 1) [33]. Upwind evacuation can remove victims from a radioactive cloud and decrease the total dose of radiation received. Removal of exposed clothing and decontamination with municipal (tap) water will result in elimination of up to 90 percent of the radiation dose. External wounds should be covered to prevent contamination of first responders and medical staff, Assume wounds are contaminated by radiation, and irrigate with water. Use a radiation surveymeter after each irrigation to assess effective decontamination. After decontamination, coverwounds with waterproof dressings or primary closure when possible. The presence of vomiting within four hours, a rapid decrease in absolute lymphocyte count, or a rising neutrophil to lymphocyte ratio can be used to estimate potential exposure and prognosis [34,35]. The use of oral potassium iodide (130 mg daily for adults, 65 mg for children 3 to 18 years old, 32 mg for children 1 month to 3 years old and 16 mg for children under 1 month old) prevents accumulation of radioactive Iodine-131 in the thyroid and long-term neoplastic risk if given promptly within the first few hours of exposure [36]. Initial management is supportive with neutropenic precautions, intravenous fluids and hemodynamic support. In latent phase individuals, colony stimulating factor and blood product transfusion may be required to support pancytopenia. Amifostine, a US Food and Drug Agency approved cytoprotectant for radiation injury is unlikely to be feasibly deployed given cost and significant adverse events like hypotension and anaphylaxis [37,38].

## An approach to the poisoned patient in wartime

Evaluating a potential toxic exposure during wartime should prioritize [1] immediate lifesaving interventions and resuscitation [2], evacuation from the exposure [3], diagnosis of syndromic symptoms or toxidromes, and [4] supportive care (Figure 1). For agents with reversal agents, administration of antidotal therapy as proximal to the exposure (such as in the prehospital setting or battlefield) may increase survival and decrease the triage of scarce critical equipment (ventilators, intravenous fluids), medical staff (intensivists or critical care nurses) or space (hospital beds) in an already strained healthcare system. First responders at the scene of a potential CBRN attack should don personal protective equipment (PPE) when possible to avoid secondary contamination. Toxic exposure results in characteristic physical examination findings which may guide the administration of antidotes and other life-saving treatments [39]. We recommend basing this on physical examination findings performed in the field, with initiation of treatment as early as possible, ideally in the prehospital setting. For example, first responders used the presence of miosis to detect individuals who needed further evaluation at the scene of the sarin gas attack in Matsumoto, Japan [40]. Importantly, for individuals exposed to weaponized organophosphates and radioactive particles, decontamination should occur outside the hospital in order to prevent secondary exposures to healthcare workers and other patients. Triage should be performed by staff equipped with appropriate level PPE to perform life saving measures consistent with Sort, Assess, Life-saving interventions, Treat/Transport (SALT) triage methods or similar disaster triage algorithm [41]. Once immediate critical care and decontamination are complete, secondary triage can be performed to continuously match clinical needs with available resources to balance demand of large numbers of incoming patients.

## Conclusion

The ongoing war in Ukraine has already resulted in one of the largest humanitarian crises in the world since World War II. Unlike other recent conflicts in Libya, Afghanistan, and Yemen, the prospect of chemical, radiological and pharmaceutical-based weapons being used by state actors is higher than ever before. While the use of chemical weapons has been internationally prohibited by the Chemical Weapons Convention in 1997, advances in the manufacturing of chemical weapons and an increase in their use in conflict zones in the recent decade raise concern of their use during the Ukraine-Russia war [16,42]. It is critical to consider strategies to best recognize and provide toxicological first aid to individuals exposed to these weapons of mass destruction. The Russian invasion of Ukraine highlights the need to prepare for offensive use of chemical weapons or radioactive material against soldiers and civilians. We hope that an early end of this war will preclude their use.
